# Tackling imported tropical diseases in China

**DOI:** 10.1038/s41426-018-0022-4

**Published:** 2018-02-07

**Authors:** Xiao-Nong Zhou, Men-Bao Qian, Gerardo Priotto, José Ramón Franco, Jia-Gang Guo

**Affiliations:** 1 Key Laboratory on Biology of Parasite and Vector, Ministry of Health, Shanghai, 200025 China; 2 National Center for International Research on Tropical Diseases, Shanghai, 200025 China; 3 WHO Collaborating Center for Tropical Diseases, Shanghai, 200025 China; 4 National Institute of Parasitic Diseases, Chinese Center for Disease Control and Prevention, Shanghai, 200025 China; 5 Department of Control of Neglected Tropical Diseases, World Health Organization, Geneva, 1211 Switzerland

Tropical diseases predominantly occur in the tropics. They are often referred to as infectious diseases that thrive in hot and humid conditions^1^. Owing to rapid economic development and massive control activities, significant achievements have been realized in the control of tropical diseases in China. Typical examples include the elimination of lymphatic filariasis in 2007 and ambitious targets to eliminate malaria and schistosomiasis by 2020 and 2025, respectively^2^. However, new challenges have also risen from economic development in China and Sino-Foreign cooperation triggered by the “Belt and Road Initiative”, which has promoted the international movement of a huge population. Approximately 60 million Chinese people live overseas^3^. Another one million Chinese migrant workers participated in temporary employment abroad in 2014 under the Chinese Ministry of Commerce’s International Labor Cooperation program^3^. Furthermore, the number of Chinese people traveling overseas reached 122 million in 2016^4^. Additionally, 52.67 million foreigners crossed China’s border in 2014^3^. Economic globalization and population migration have increased the importation risk of tropical diseases that were not endemic in China.

Human African trypanosomiasis (HAT) is a neglected tropical disease that affects 36 African endemic countries and has been targeted for elimination by the World Health Organization (WHO). In the last 15 years, important advances have been made in controlling this disease, and a substantial, steady reduction in the number of cases has been observed, reaching the historical lowest figure (2184 cases) in 2016^5, 6^. Sporadic cases are diagnosed in non-endemic countries among travelers (e.g., tourists, workers, and migrants)^7^. Last August, two HAT cases were detected in China^8, 9^. The first HAT case was detected in China in 2014^10^, and these three HAT cases urgently directed our attention towards imported tropical diseases that could be lethal without prompt and proper treatment. The first and third cases were infections with *Trypanosoma brucei gambiense*, and these individuals were presumably infected in Gabon, Western Africa^9, 10^. The second case was caused by *T. b. rhodesiense* with a probable infection in Tanzania, Eastern Africa^8^. The former two cases were discovered in exported Chinese workers, whereas the second case was found in a Chinese tourist visiting game parks. Due to the acute presentation of *rhodesiense* HAT, the second case received diagnosis and treatment within 1 month. However, the two *gambiense* HAT cases underwent long, delayed diagnosis, and the disease was in the second stage (invasion of the central nervous system) when a definite diagnosis was reached. Thus, delayed diagnosis increases the challenge of subsequent treatment.

These three HAT cases exposed weaknesses in controls against imported tropical diseases in China. First, the workers and travelers had no awareness of prevention and protection against tropical diseases in endemic countries. They had no awareness; health education on imported tropical diseases is limited, and counseling services are inadequate, particularly for non-epidemic diseases in China. Second, due to the rarity of these imported tropical diseases, medical organizations lack diagnostic capacity, which usually causes a significant delay in diagnosis. Third, the unavailability of specific medicines in China complicated treatment initiation. Although the efforts and coordination required to successfully obtain specific drugs supplied by WHO through the WHO Collaborating Center for Tropical Diseases at the National Institute of Parasitic Diseases affiliated to the Chinese Center for Disease Control and Prevention should be highly commended, constraints in the transportation and delivery of these medicines created an additional risk for patients.

Thus, all these factors demonstrate that attention to imported tropical diseases is urgently needed in China. Corresponding to the aforementioned weaknesses, specific measures should be implemented. First, a database of imported tropical diseases in China should be established. The database should include tropical diseases that have already been imported into China and those that have not yet been imported but may be potentially imported in the future. Second, medical organizations and entry-exit administrative departments should establish a mechanism for medical counseling services for overseas workers and travelers. Third, diagnostic capacity for imported tropical diseases should be strengthened, which includes instruction in medical schools and the training of special doctors in hospitals. Training could be provided through WHO Collaborating Centers in China. Finally, a mechanism to provide specific drugs with fast channels for prompt treatment of imported and rare tropical diseases should be constructed, and it should include at minimum aspects such as (i) risk evaluation of imported tropical diseases; (ii) establishment of a medicine stock for tropical diseases in China; (iii) a management mechanism for this stock in coordination with WHO; and (iv) case reports and drug supply mechanisms.
